# Analyzing stability parameters for assessing immediate and early loading of implants

**DOI:** 10.6026/973206300212807

**Published:** 2025-08-31

**Authors:** Sachin B. Mangalekar, Rohit Sharma, Mohammad Jalaluddin, Purnima Nadkerny, Mudambi Prakash Pavani, Pramod V., Miral Mehta

**Affiliations:** 1 Department of Periodontology, Bharati Vidyapeeth (Deemed to be University) Dental College and Hospital, Wanlesswadi, Sangli-416414, Maharashtra, India; 2 Department of Dentistry, Amaltas Institute of Medical Sciences, Dewas, Madhya Pradesh, India; 3 Department of Periodontics and Implantology, Kalinga Institute of Dental Sciences, KIIT Deemed University, Bhubaneswar-751024, India; 4 Department of Periodontology, Daswani Dental College and Research Centre, Kota, Rajasthan, India; 5 Department of Periodontology, Meghna Institute of Dental Sciences, Nizamabad, Telangana, India; 6 Department of Pediatric and Preventive Dentistry, Karnavati School of Dentistry, Karnavati University, Gandhinagar, Gujarat, India

**Keywords:** Implant stability, immediate loading,, early loading, ISQ, resonance frequency analysis, insertion torque, osseointegration

## Abstract

Immediate and early loading of dental implants reduces treatment time and enhances patient satisfaction, but success depends on achieving sufficient primary stability and early osseointegration. This clinical trial compared implant stability parameters for guiding loading protocols in 40 patients requiring single posterior mandibular implants. Resonance Frequency Analysis (ISQ), Periotest values (PTV) and insertion torque were recorded at placement and re-evaluated at 3 weeks and 3 months, alongside radiographic bone loss assessment. Both groups showed progressive ISQ improvement and minimal marginal bone loss, with no significant differences between immediate and early loading. Thus, we show that ISQ and insertion torque are reliable indicators for safe loading decisions in implant therapy.

## Background:

Dental implants have become a widely accepted treatment modality for replacing missing teeth due to their high success rates and ability to restore function and esthetics [1]. Traditionally, implants were subjected to a healing period of three to six months before prosthetic loading to allow for osseointegration, as proposed by Brånemark. However, advances in implant design, surface modifications and surgical techniques have enabled the development of immediate and early loading protocols, significantly reducing treatment time and improving patient comfort [2]. The success of immediate and early loading primarily depends on the achievement of adequate primary stability at the time of implant placement and the maintenance or enhancement of stability during the early healing phase. Primary stability is influenced by bone density, surgical technique and implant geometry, whereas secondary stability is the result of biological integration of the implant with bone tissue [3, 4]. Various methods have been developed to assess implant stability, including insertion torque measurement, resonance frequency analysis (RFA) and Periotest values. These non-invasive techniques offer valuable insights into the biomechanical and biological behavior of implants under functional loading [5, 6]. Resonance frequency analysis (RFA), quantified using Implant Stability Quotient (ISQ) values, is widely employed to monitor implant stability over time. Studies have shown that ISQ values above 65-70 may indicate sufficient stability for early or immediate loading protocols [7, 8]. Similarly, insertion torque values greater than 35 Ncm are often used as a clinical benchmark for immediate loading feasibility [9]. Despite the availability of these diagnostic tools, there remains variability in defining threshold values and their predictive accuracy regarding implant success under different loading protocols [10]. Immediate loading refers to placing the prosthetic restoration within 48 hours of implant insertion, whereas early loading typically occurs within 3-6 weeks post-placement. While these approaches offer significant benefits in terms of patient satisfaction and reduced treatment time, they also carry the risk of micromotion at the bone-implant interface, which may jeopardize osseointegration if not adequately controlled [3, 11]. Therefore, it is crucial to carefully evaluate implant stability at multiple time points to ensure safe loading and long-term success. The concept of "stability dip," which represents a temporary reduction in stability during the early healing phase, further emphasizes the need for precise monitoring [12]. Insertion torque is one of the primary indicators used during surgical placement to assess mechanical engagement of the implant with surrounding bone. High insertion torque suggests good primary stability, especially in dense bone (Type I or II), but excessive torque may cause microfractures, leading to bone resorption [4]. Torque values between 35-45 Ncm are commonly considered optimal for immediate or early loading protocols. However, relying solely on torque values may be insufficient, as they reflect only the initial mechanical engagement and not the biological stability that develops post-placement [5].

Resonance Frequency Analysis (RFA) provides a dynamic evaluation of implant stability by assessing micromovements at the implant-bone interface. It is a repeatable, non-invasive tool that helps clinicians track changes in implant stability during the healing period [6]. RFA values are expressed as Implant Stability Quotients (ISQ), which range from 1 to 100. ISQ values greater than 70 are often associated with successful outcomes in immediate and early loading protocols, although this threshold may vary depending on implant system, bone quality and site of placement [7, 8]. Serial measurements of ISQ help detect early signs of implant failure and guide the timing of prosthetic loading. Periotest, another tool for assessing implant stability, measures the damping characteristics of the peri-implant tissues and provides a quantitative assessment of implant mobility. Unlike RFA, which evaluates axial stability, Periotest values (PTVs) offer insight into lateral mobility. A more negative PTV indicates higher stability. While less commonly used than RFA, Periotest has shown potential in supplementing clinical decisions regarding implant loading, especially when combined with other parameters [9, 10]. Overall, a multimodal approach to stability assessment may enhance the clinician's ability to safely adopt immediate or early loading strategies. Therefore, it is of interest to analyze and compare key stability parameters-namely ISQ values, insertion torque and Periotest readings-between immediate and early loading groups. Understanding the correlation between these parameters and clinical outcomes will help refine clinical guidelines for safe and predictable loading of implants.

## Materials and Methods:

This prospective clinical study was conducted on 40 systemically healthy patients aged between 25 and 55 years who required single-tooth implants in the posterior mandible. Participants were selected based on specific inclusion and exclusion criteria. Inclusion criteria included adequate bone volume for implant placement without the need for augmentation, absence of periodontal disease and willingness to follow post-operative protocols. Patients with systemic conditions affecting bone healing, smokers and those with parafunctional habits were excluded.

## All patients were randomly divided into two equal groups:

[1] Group A (Immediate Loading, n = 20): Implants were loaded with a provisional crown within 48 hours of placement.

[2] Group B (Early Loading, n = 20): Implants were loaded after a healing period of three weeks post-placement.

Surgical procedures were performed under local anesthesia using a flapless or minimal flap technique based on preoperative CBCT evaluation. Implants with a diameter of 4.0 mm and a length of 10-12 mm were placed using a standardized drilling protocol.

## Primary implant stability was assessed at placement using:

[1] Insertion Torque: Measured in Newton centimeters (Ncm) using a calibrated torque wrench.

[2] Resonance Frequency Analysis (RFA): Measured using an Osstell^TM^ device, with values recorded in Implant Stability Quotient (ISQ).

[3] Periotest Values (PTV): Measured using the Periotest M device, with lower values indicating greater stability.

Follow-up evaluations were conducted at baseline, 3 weeks and 3 months. RFA and PTV were repeated at each time point. Radiographic assessment was carried out using standardized digital periapical radiographs to evaluate marginal bone levels at baseline and after 3 months. Bone loss was measured from the implant shoulder to the first bone-to-implant contact using image analysis software. All data were statistically analyzed using SPSS version 25.0. Intergroup comparisons were performed using independent t-tests, while intragroup changes over time were analyzed using paired t-tests. A p-value < 0.05 was considered statistically significant.

## Results:

A total of 40 implants were placed and monitored throughout the study, with all implants successfully osseointegrated by the 3-month follow-up. Clinical parameters including insertion torque, ISQ values, Periotest readings and marginal bone loss were recorded and analyzed. At the time of placement, Group A (Immediate Loading) exhibited a higher mean insertion torque of 42.5 ± 4.1 Ncm, while Group B (Early Loading) had a mean of 38.6 ± 3.9 Ncm. This difference was statistically significant (p < 0.05) (Table 1 - see PDF). In terms of ISQ values, Group A showed a baseline mean of 73.2 ± 2.5, which increased to 75.1 ± 2.1 at 3 weeks and 77.4 ± 1.8 at 3 months. Group B started with a mean ISQ of 70.8 ± 3.0, increasing to 73.7 ± 2.4 at 3 weeks and 76.2 ± 2.0 at 3 months. Intergroup comparison showed no statistically significant difference at 3 months (p = 0.087) (Table 2 - see PDF). Periotest Values (PTV) was also measured at each interval. At baseline, Group A recorded a mean PTV of -2.1 ± 0.5, while Group B showed -1.7 ± 0.6. At 3 months, the values improved to -3.0 ± 0.4 and -2.8 ± 0.3 respectively, with no significant intergroup difference noted (p = 0.131) (Table 3 - see PDF). Radiographic analysis revealed minimal marginal bone loss in both groups. At the 3-month follow-up, Group A demonstrated a mean bone loss of 0.52 ± 0.08 mm, while Group B showed 0.48 ± 0.07 mm. The difference was not statistically significant (p = 0.211) (Table 4 - see PDF). Overall, both loading protocols demonstrated comparable improvements in stability metrics and bone preservation, with minor differences at various time points (Tables 1-4 - see PDF).

## Discussion:

This study evaluated the stability parameters-namely insertion torque, ISQ values from resonance frequency analysis (RFA) and Periotest readings-for assessing the feasibility of immediate and early loading of dental implants. The results indicated that both protocols demonstrated favorable outcomes in terms of implant stability and marginal bone preservation, with no statistically significant differences between the two groups by the end of 3 months. Primary stability, a critical factor for the success of early and immediate loading protocols, is primarily determined by mechanical engagement between the implant and the surrounding bone at the time of placement [1, 2]. In our study, Group A (immediate loading) had significantly higher insertion torque values than Group B (early loading), suggesting more favorable bone quality or implant engagement in these cases. Studies have consistently shown that insertion torque values above 35 Ncm are predictive of successful immediate loading [3, 4]. However, excessively high torque (>50 Ncm) can lead to microfractures and impaired osseointegration [5]. Resonance frequency analysis (RFA) is widely accepted as a reliable method for assessing implant stability over time [6, 7]. In this study, ISQ values in both groups progressively increased, indicating successful osseointegration. Group A showed slightly higher ISQ values at all-time points, although the differences were not statistically significant at 3 months. These findings are in line with previous studies where ISQ values above 70 were associated with predictable outcomes in immediately loaded implants [8, 9]. It has also been reported that an increase in ISQ values over time is a positive sign of biological stability replacing initial mechanical anchorage [10]. Periotest values (PTV), though less commonly used than RFA, provide additional information on implant micromobility. Lower (more negative) PTVs correlate with higher stability. Our findings revealed a trend of improved PTV scores in both groups over time, supporting the ISQ findings. Although some authors argue that PTV may be influenced by surrounding soft tissue characteristics, when used in conjunction with RFA, it adds depth to the overall stability assessment [11, 12]. Marginal bone loss is another crucial parameter reflecting long-term success and biological integration. In our study, both groups exhibited minimal bone resorption (<0.6 mm), well within the acceptable threshold of 1.5 mm in the first year, as proposed by Albrektsson *et al.* [13]. Several studies have reported comparable bone loss outcomes in immediate and early loading protocols when appropriate case selection and primary stability are ensured [14, 15]. These findings support the view that accelerated loading can be safely adopted without compromising osseointegration if biomechanical and biological criteria are met.

## Conclusion:

The value of multimodal implant stability assessment is shown. RFA, insertion torque and PTV together provide a comprehensive picture that aids in making evidence-based decisions regarding implant loading. Further long-term, multicenter trials are necessary to establish standardized threshold values for different bone types and implant systems.
